# Three New Tetranorditerpenes from Aerial Parts of Acerola Cherry (*Malpighia emarginata*)

**DOI:** 10.3390/molecules19022629

**Published:** 2014-02-24

**Authors:** Jie-Qing Liu, Yuan-Yuan Deng, Ting-Zhao Li, Qiang Han, Yan Li, Ming-Hua Qiu

**Affiliations:** 1 State Key Laboratory of Phytochemistry and Plant Resources in West China, Kunming Institute of Botany, Chinese Academy of Science, Kunming 650201, China; E-Mails: liujieqing@mail.kib.ac.cn (J.-Q.L.); yuanyuan_deng@yeah.net (Y.-Y.D.); liyanb@mail.kib.ac.cn (Y.L.); 2 University of Chinese Academy of Science, Beijing 100049, China; 3 Amway (China) Botanical Research Center, Wuxi 214115, China; E-Mails: teric.li@amway.com (T.-Z.L.); johnson.han@amway.com (Q.H.)

**Keywords:** acerola, *Malpighia emarginata*, Malpighiaceae, tetranorditerpenes, cytotoxicity

## Abstract

Acerola cherry is a world famous fruit which contains abundant antioxidants such as vitamin C, anthocyanins, flavonoids, and phenolics. However, studies concerning bioactivity components from aerial parts of acerola (*Malpighia emarginata*) are scarce. In view of this, we have examined the constituents of aerial parts of acerola, and three new tetranorditerpenes acerolanins A–C (**1**–**3**) with a rare 2*H*-benz[*e*]inden-2-one substructure were isolated. Their structures were determined on the basis of spectral studies and acerolanin C was confirmed by X-ray crystallographic analysis. Furthermore, three new compounds have been studied for their cytotoxic activity.

## 1. Introduction

Acerola (*Malpighia emarginata* DC.) is a shrub grown in tropical and subtropical areas. It has been introduced into many provinces of China including Guangxi, Guangdong, and Yunnan, *etc*. Acerola fruits are mainly utilized by the supplement, pharmaceutical, and fruit-juices industries as a rich source of vitamin C [1]. However, recent research showed that besides vitamin C, acerola fruits may be also a good source of phytochemicals such as anthocyanins [2,3], flavonoids and phenolic acids [4], and polyphenols [2,5]. With respect to bioactivities, acerola showed antioxidant [6], antimicrobial [7], hepatoprotective [8], and anti-hyperglycemic [9] effects. Nevertheless, there is seldom report on the bioactivity constituents from aerial parts of acerola.

**Figure 1:**
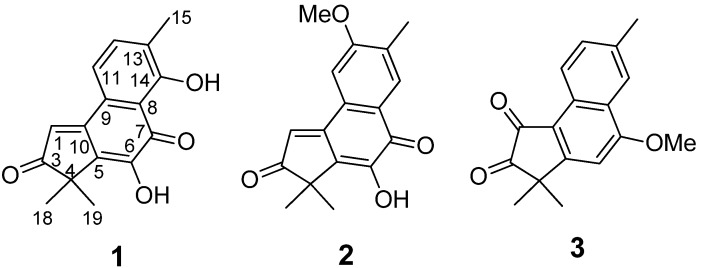
Structures of compounds **1**–**3** from the aerial parts of acerola.

In our previous research, three norfriedelins with acetylcholinesterase inhibitory activity were found in acerola tree (*M.* *emarginata*) [10]. As continuation of this work, we have examined the lipophilic constituents of aerial parts of acerola collected in Nanning, Guangxi Province, China. Three new tetranorditerpenes (**1**–**3**) with a rare 2*H*-benz[*e*]inden-2-one substructure were obtained (Figure 1). This report describes the isolation and structural determination of the compounds, as well as their cytotoxic activities.

## 2. Results and Discussion

**Figure 2:**
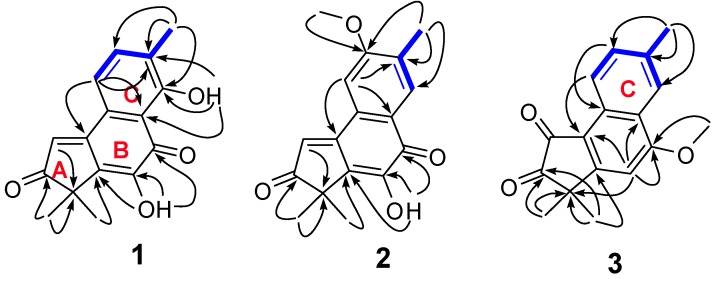
The key HMBC (H→C) and ^1^H-^1^H COSY correlations (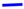) of **1**–**3**.

Compound **1** was obtained as yellow power and had a molecular formula C_16_H_14_O_4_ by HREI-MS ion at *m/z* 270.0900 [M]^+^ with 10 degrees of unsaturation. ^13^C-DEPT (Table 1) revealed sixteen resonances consisting of two carbonyls, ten olefinic carbons, one quaternary carbon and three methyl groups. Seven out of ten degrees of unsaturation were occupied by two carbonyls and five double bonds and the remaining three indicated that compound **1** was tricyclic. The 1D NMR data of **1** was similar to those of substructure B in fimbricalyx A [11]. In the HMBC spectrum (Figure 2), cross-peaks between δ_H_ 1.44 (H_3_-18 and H_3_-19) and δ_C_ 209.4 (C-3), 46.2 (C-4), and 133.6 (C-5) suggested that the carbonyl was located at C-3. According to the HSQC spectrum, two hydroxyl proton signals were identified at δ_H_ 7.27 and 12.08. Correlations from δ_H_ 7.27 to 133.6 (C-5), 141.0 (C-6), and 184.6 (C-7), from δ_H_ 12.08 to 112.9 (C-8), 131.5 (C-13), and 160.9 (C-14) in the HMBC spectrum suggested that two hydroxyl groups were attached to C-6 and C-14, respectively. Two aromatic proton signals at δ_H_ 7.41 (1H, d, *J* = 6.0 Hz) and δ_H_ 7.45 (1H, d, *J* = 6.0 Hz) were attributed to H-11 and H-12, respectively, by their correlations with each other in the ^1^H-^1^H COSY spectrum and by the HMBC correlations from δ_H_ 7.41 to C-13, C-8, and C-10, from δ_H_ 7.45 to C-14 and Me-15. The formation of an intramolecular hydrogen bond between 14-OH with 7-(C=O) shifted the proton signal of 14-OH to lower field at δ_H_ 12.08, which further confirmed the C-7 location of the carbonyl group. Thus, the structure of compound **1**, named acerolanin A, was identified as shown in Figure 1.

**Table 1:** ^1^H- and ^13^C-NMR data for compounds **1**–**3** (in CDCl_3_, 600 MHz for ^1^H and 150 MHz for ^13^C, δ in ppm).

No.	1		2		3	
	δC	δH (mult, *J* in Hz)	δC	δH (mult, *J* in Hz)	δC	δH (mult, *J* in Hz)
1	125.9, d	6.77 (s)	125.2, d	6.76 (s)	184.3, s	
3	209.4, s		209.9, s		205.6, s	
4	46.2, s		46.3, s		43.2, s	
5	133.6, s		131.0, s		163.4, s	
6	141.0, s		141.3, s		98.7, d	6.80 s
7	184.6, s		180.1, s		164.6, s	
8	112.9, s		130.7, s		133.2, s	
9	128.5, s		131.9, s		128.8, s	
10	118.4, s		156.3, s		125.2, s	
11	155.6, d	7.41 (d, 6.0)	106.5, d	7.25 (s)	124.8, d	9.05 (d, 8.4)
12	136.7, d	7.45 (d, 6.0)	162.1, s		133.1, d	7.62 (d, 8.4)
13	131.5, s		122.8, s		137.9, s	
14	160.9, s		129.8, s	8.02 (s)	122.4, d	8.12 (s)
15	16.1, q	2.35 (s)	16.8, q	2.33 (s)	22.1, q	2.56 (s)
18	21.6, q	1.44 (s)	21.7, q	1.44 (s)	25.0, q	1.50 (s)
19	21.6, q	1.44 (s)	21.7, q	1.44 (s)	25.0, q	1.50 (s)
-OMe			56.0, q	4.00 (s)	56.6, q	4.20 (s)
6-OH;		7.27 (s)		7.01 (s)		
14-OH		12.08 (s)				

Compound **2** was obtained as a light yellow power and had a molecular formula C_17_H_16_O_4_ as evidenced by HREI-MS at *m*/*z* 284.1056 [M]^+^. The 1D NMR data (Table 1) of **2** showed high similarities to those of **1** except that there was one methoxyl group (δ_C_ 56.0 and *δ*_H_ 4.00) in **2** instead of one hydroxyl group in **1**. The methoxyl group was determined to be attached to C-12 by the following evidence: HMBC (Figure 2) correlations from *δ*_H_ 2.33 (15-CH_3_) to δ_C_ 129.8 (C-14), 162.1 (C-12), and 122.8 (C-13), from δ_H_ 4.00 (MeO-12) to δ_C_ 162.1 (C-12); Furthermore, the fact that δ_H_ 7.25 (H-11, 1H) and 8.02 (H-14, 1H) were singlets in the ^1^H-NMR demonstrated they were not in the *ortho*-position as in compound **1**. Further detailed study of the HMBC and ^1^H-^1^H COSY data (Figure 2) determined the other parts of the structure of **2**. Therefore, the structure of compound **2** was elucidated as shown in Figure 1. The compound was named acerolanin B.

**Figure 3:**
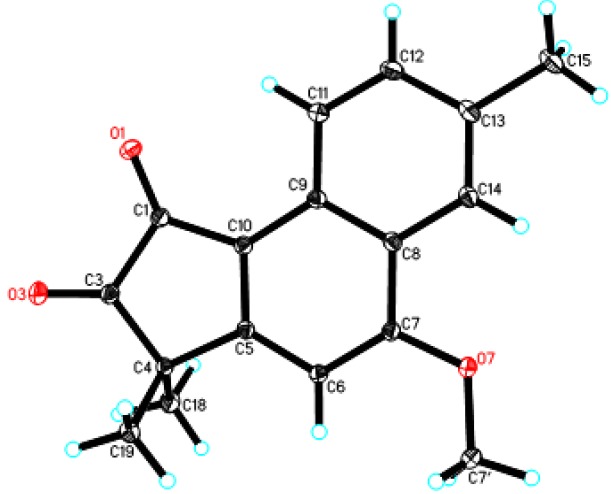
X-Ray crystal structure of **3**.

Acerolanin C (**3**) was obtained as colorless monoclinic crystals from CHCl_3_/MeOH (1:3). The molecular formula C_17_H_16_O_3_ was established by the positive HREI-MS (found [M]^+^ at *m*/*z* 268.1097, calcd for C_17_H_16_O_3_ at *m*/*z* 268.1099), corresponding to 10 degrees of unsaturation. The skeleton of **3** was the same as **2** according to its 1D NMR data (Table 1). The difference between **3** and **2** was the absence of one hydroxyl group in **3** according to comparison of their formula and ^13^C-DEPT data (four =CH in **3** and three =CH in **2**). As supported by the HMBC correlations from Me-15 to C-12, C-13, and C-14 and by the ^1^H-^1^H COSY between H-11 (9.05, d, *J* = 8.4 Hz) with H-12 (7.62, d, *J* = 8.4 Hz), there was no substituent at C-11, C-12, and C-14 Thus, the only methoxyl group could be located at C-7 by the HMBC correlations from H-6 to C-4, C-8, and C-10, from MeO-7 to C-7. In the HMBC spectrum, two methyl-proton signals at δ_H_ 1.50 correlated with a carbonyl-carbon signal at δ_C_ 205.6 suggested one carbonyl at C-3. Therefore, the other carbonyl should be located at C-1. In order to confirm its structure, the X-ray crystallography of **3** was completed and the result (Figure 3) allowed unambiguous assignment of its planar structure.

Acerolanins A–C are a class of tetranorditerpenoids possessing a rare 2*H*-benz[*e*]inden-2-one substructure. Analogues have been isolated previously from four species of the Euphorbiaceae, namely, *Neoboutonia glabrescens* [12], *Neoboutonia mannii* [13], *Trigonostemon howii* [14], and *Strophioblachia fimbricaly* [11]. Some compounds of this class were reported to have cytotoxic and antimicrobial activities, so compounds **1**–**3** were evaluated for cytotoxicity against HL-60, SMMC-7721, A-549, MCF-7, and SW480 human tumor cell lines using the MTS method. Cisplatin was used as a positive control. Results are summarized in Table 2. As can be observed, compounds **1**–**3** showed moderate cytotoxicity against above five cell lines with IC_50_ values from 10 to 40 μM.

**Table 2:** Cytotoxicity data of compounds **1−3** with IC_50_ values (μM) *^a^*.

Compounds	HL-60	SD	SMMC-7721	SD	A-549	SD	MCF-7	SD	SW480	SD
**1**	10.23	0.32	12.20	0.42	12.32	0.45	16.22	0.72	18.12	0.86
**2**	14.11	0.61	16.54	0.63	18.27	0.72	22.08	1.12	24.32	1.21
**3**	22.17	1.80	20.10	1.07	31.65	1.22	28.04	1.47	>40	-
**Cisplatin**	1.86	0.10	6.13	0.34	7.27	0.42	15.27	0.65	16.23	0.76

*^a^* Data were obtained from triplicate experiments, and cisplatin was used as positive control. SD = standard deviation.

## 3. Experimental

### 3.1. General Procedures

^1^H- and ^13^C-NMR spectra were measured on Bruker AVANCE III-600 instruments (Bruker, BioSpin International AG, Karlsruhe, Germany) with trimethylsilane (TMS) as the internal standard. ESIMS were recorded on a VG Auto Spec-3000 mass spectrometer (VG, Manchester, UK), while HREIMS was measured on an AutoSpec Premier P776 mass spectrometer (Water Corporation, Billerica, MA, USA). IR spectra were obtained on a Bio-Rad FTS-135 spectrometer (Bio-Rad Laboratories Inc., Richmond, CA, USA).

TLC was performed on precoated TLC plates (200–250 μM thickness, F254 Si gel 60, Qingdao Marine Chemical, Inc., Qingdao, China) with compounds visualized by spraying the dried plates with 10% aqueous H_2_SO_4_ followed by heating until dryness. Silical gel (200–300 mesh, Qingdao Marine Chemical, Inc.) and Lichroprep RP-18 (40–63 μm, Merck, Darmstadt, Germany) were used for column chromatography. Methanol, trichloromethane, *n*-hexane, and acetone were purchased from Tianjing Chemical Reagents Co. (Tianjing, China).

### 3.2. Plant Material

The aerial parts of acerola (*Malpighia emarginata*) were collected in May 2012 from Nanning, Guangxi province, China. The samples were identified by Prof. Zongyu Wang, Kunming Institute of Botany, Chinese Academy of Science. A voucher specimen (No. KIB 2012-04-20) has been deposited at the State Key Laboratory of Phytochemistry and Plant Resources in West China, Kunming Institute of Botany, Chinese Academy of Sciences.

### 3.3. Extraction and Isolation

The powder and dried aerial parts of *M.* *emarginata* (10 kg) were extracted with acetone at room temperature (20 L × 3, 3 days each time) and concentrated *in vacuo* to give a crude extract (400 g), which was then partitioned in succession between H_2_O and CHCl_3_. The CHCl_3_ fraction (85 g) was chromatographed on silica gel with CHCl_3_/MeOH gradient elution (100:0→20:1) to afford four fractions: Fr.1 (35 g, 100:0), Fr. 2 (12 g, 100:1), Fr.3 (10 g, 50:1), and Fr.4 (25 g, 10:1). By reverse-phase silica gel (MeOH/H_2_O, step gradients), Fr.2 was divided into three parts (MeOH/H_2_O: 40%, 60%, 80%), and 60% part was on chromatography over silica gel with *n*-hexane/CHCl_3_ (2:1) to yield acerolanin C (**3**, 7 mg). Using reverse-phase silica gel (MeOH/H_2_O, step gradient), Fr.3 was also divided into three fractions (MeOH/H_2_O: 40%, 60%, 80%). 60% fraction was further chromatographed on a silica gel column gradient eluting with CHCl_3_/acetone (15:1) to obtain acerolanin B (**2**, 5 mg), while 40% fraction was subjected to silica gel column chromatography (CHCl_3_/MeOH, 30:1) to give acerolanin A (**1**, 8 mg).

### 3.4. Characteristic Data of Compounds **1**–**3**

*Acerolanin A* (**1**). Yellow powder; UV in CHCl_3_ λ_max_ (log ε): 444 (4.86), 326 (4.98), 239 (4.88). IR (KBr) *v*_max_ 3450, 1680, 1601, 1311, 1236 cm^−1^; ^1^H- and ^13^C-NMR data: see Table 1; positive ESI-MS: *m/z* 293 [M + Na]^+^; positive HREIMS: *m/z* 270.0900 [M]^+^ (calcd for C_16_H_14_O_4_, 270.0892).

*Acerolanin B* (**2**). Light yellow powder; UV in CHCl_3_ λ_max_ (log ε): 345 (4.52), 275 (4.51), 230 (4.23). IR (KBr) *v*_max _3461, 1683, 1637, 1597, 1383, 1270, 1247 cm^−1^; ^1^H- and ^13^C-NMR data: see Table 1; positive ESI-MS: *m/z* 307 [M + Na]^+^; positive HREIMS: *m/z* 284.1056 [M]^+^ (calcd for C_17_H_16_O_4_, 284.1049).

*Acerolanin C* (**3**). **C**olorless Monoclinic crystals from CHCl_3_/MeOH (1:3); UV in CHCl_3_ λ_max_ (log ε): 389 (3.98), 274 (4.11), 239 (4.06). IR (KBr) *v*_max _1755, 1682, 1570, 1463, 1231 cm^−1^; ^1^H- and ^13^C-NMR data: see Table 1; positive ESI-MS: *m/z* 291 [M + Na]^+^; positive HREIMS: *m/z* 268.1097 [M]^+^ (calcd for C_17_H_16_O_3_, 268.1099).

*Crystal data for* **3**: C_17_H_16_O_3_, *M* = 268.30, monoclinic, a = 8.8643(9) Å, b = 13.7477(14) Å, *c* = 11.3957(12) Å, α = 90.00°, β = 104.141(2)°, γ = 90.00°, V = 1346.6(2) Å^3^, T = 100(2) K, space group *P*21*/c*, *Z* = 4, *μ*(MoKα) = 0.090 mm^−1^, 14026 reflections measured, 3824 independent reflections (*R_int_* = 0.0282). The final *R_1_* values were 0.0423 (*I* > 2*σ*(*I*)). The final *wR*(*F*^2^) values were 0.1020 (*I* > 2*σ*(*I*)). The final *R_1_* values were 0.0620 (all data). The final *wR*(*F*^2^) values were 0.1157 (all data). The goodness of fit on *F*^2^ was 1.026. The crystal structure of **3** was solved by direct method SHELXS-97 (Sheldrich, G.M. University of Gottingen: Gottingen, Germany, 1997) and the full-matrix least-squares calculations. Crystallographic data for the structure of **2** have been deposited with the Cambridge Crystallographic Data Centre (deposition number: CCDC 939851). CCDC 939851 contains the supplementary crystallographic data for this paper. These data can be obtained free of charge via the Internet at www.ccdc.cam.ac.uk/conts/retrieving.html (or from the CCDC, 12 Union Road, Cambridge CB2 1EZ, UK; Fax: +44 1223 336033; E-mail: deposit@ccdc.cam.ac.uk.

### 3.5. Cytotoxicity Assay

The cytotoxicity of compounds **1**–**3** was tested against human breast cancer (MCF-7), hepatocellular carcinoma (SMMC-7721), myeloid leukemia (HL-60), lung cancer (A-549) and colon cancer (SW480) cell lines using an MTS (3-(4,5-dimethylthiazol-2-yl)-5-(3-carboxymethoxyphenyl)-2-(4-sulfophenyl)-2*H*-tetrazolium inner salt) assay, with cisplatin (Sigma-Aldrich, St. Louis, MO, USA) as the positive control. All the cell lines were obtained from Shanghai cell bank in China and were cultured in RPMI-1640 or DMEM medium (Hyclone, Logan, UT, USA), supplemented with 10% fetal bovine serum (Hyclone, Logan, UT, USA) at 37 °C in a humidified atmosphere containing 5% CO_2_. The viability of cells was determined by performing colorimetric measurements of soluble formazan formed through the reduction of MTS in living cells. In brief, 100 μL medium containing 5,000 cells were plated in each wells in 96 well plates and allowed to adhere for 24 h before drug treatment, while suspension cells were seeded just before drug addition at a concentration of 1 × 10^5^ cells/mL. Cells were exposed to the test compound dissolved in dimethyl sulfoxide (DMSO) at different concentrations in triplicates at 37 °C for 48 h. At the end of the incubation, the medium were replaced with MTS medium (317 μg/mL), and then the incubation was continued for 4 h at 37 °C. The optical densities of the cell lysates were measured at 490 nm using a microplate reader (Bio-Rad Laboratories, Hercules, CA, USA). The cell viability was calculated by the following formula: cell viability (%) = (OD_sample_/OD_control_) × 100%. The IC_50_ value of each compound was calculated by Reed and Muench’s method based on the corresponding dose response curve, and data were obtained from triplicate experiments. Statistical analysis was performed using the commercially available statistical software (SPSS 11.5 for Windows, SPPS Incorporation, Chicago, IL, USA).

## 4. Conclusions

Phytochemical study of the aerial parts of acerola (*M. emarginata*) has resulted in the isolation of three new degraded diterpenes **1**–**3**. As far as we know, this is the first report of this class of degraded diterpenes from the Malpighiaceae family. In addition, compounds **1**–**3** showed cytotoxic activities.

## Supplementary Materials

1D and 2D NMR spectra of compounds **1**–**3** (S1–S15) are available as Supporting Information, Supplementary materials can be accessed at: http://www.mdpi.com/1420-3049/19/2/2629/s1.
