# Supplementary material for: Three New Tetranorditerpenes from Aerial Parts of Acerola Cherry (Malpighia emarginata)
Source: Molecules. 2014 Feb 24;19(2):2629–36. doi: 10.3390/molecules19022629 (PMC6270683; doi:10.3390/molecules19022629)

Supporting Information

Figure S1. The dose response curve of compounds **1–3** and cisplatin at each tested cell lines.

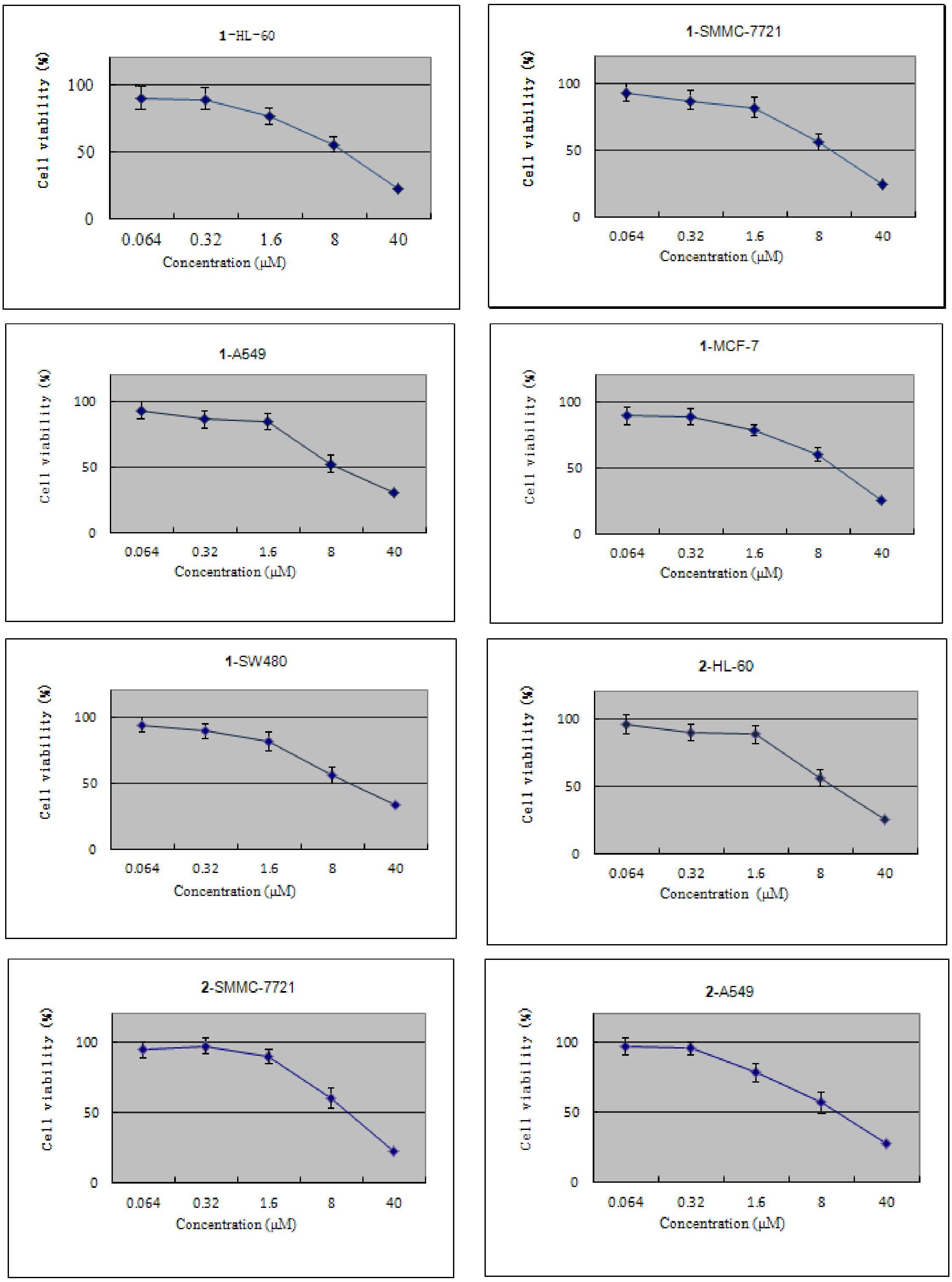

Figure S1. *Cont.*

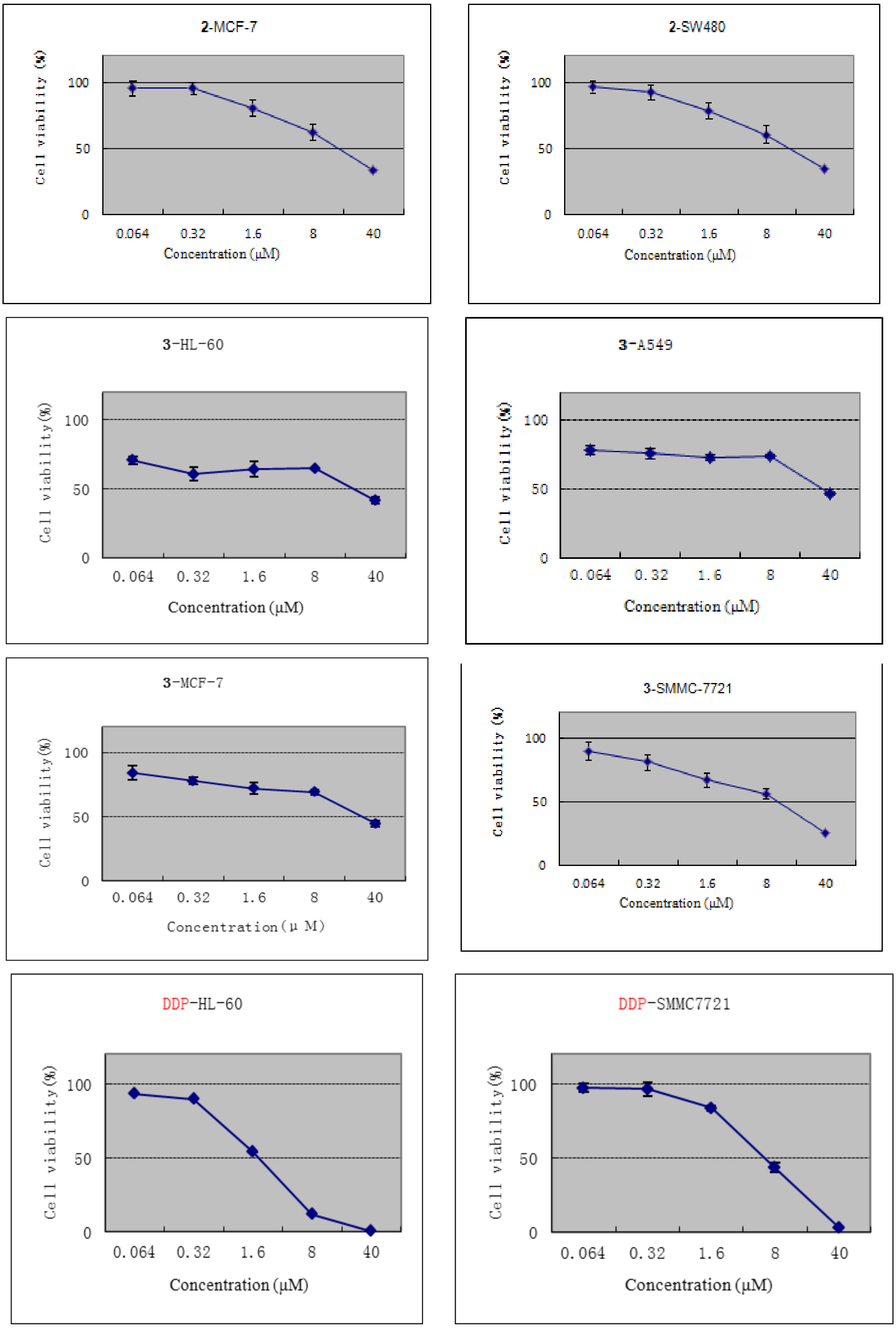

Figure S1. *Cont.*

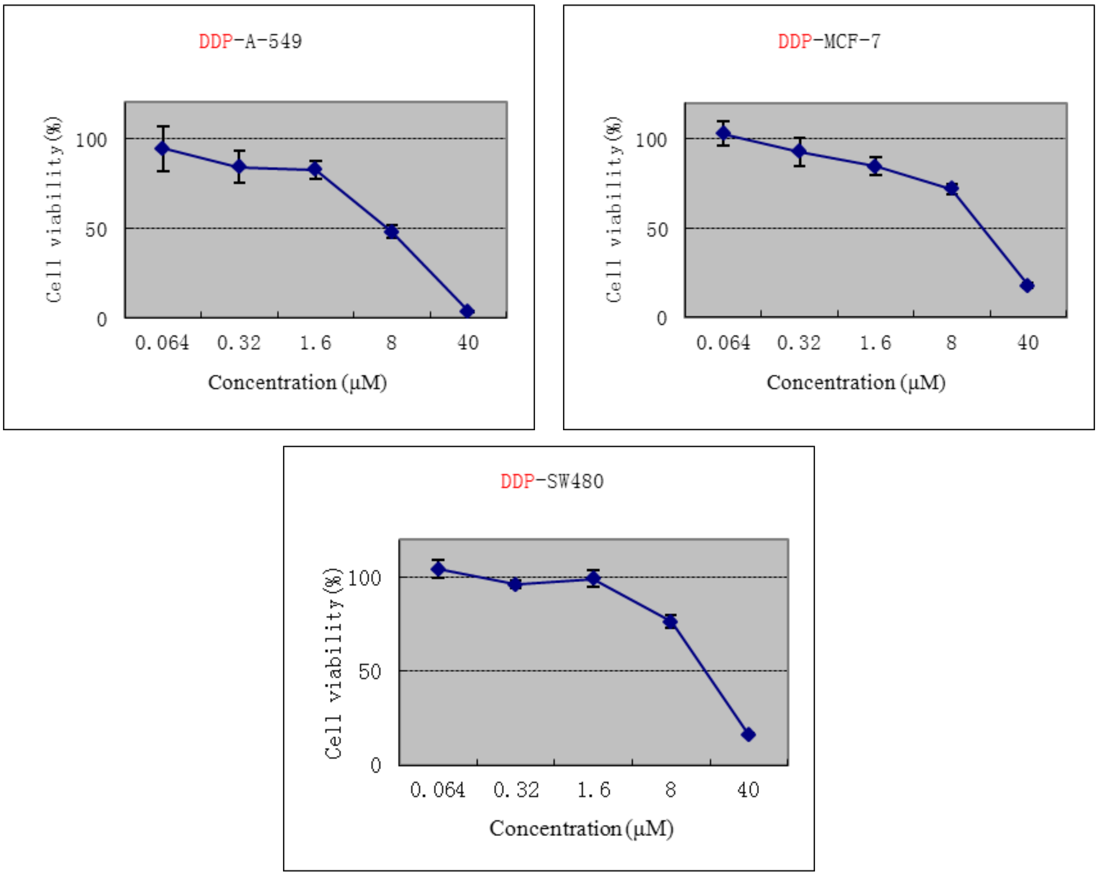

Figure S2. ^{1}H-NMR (600 Hz, $CDCl_3$) spectrum of the new compound acerolanin **A**.

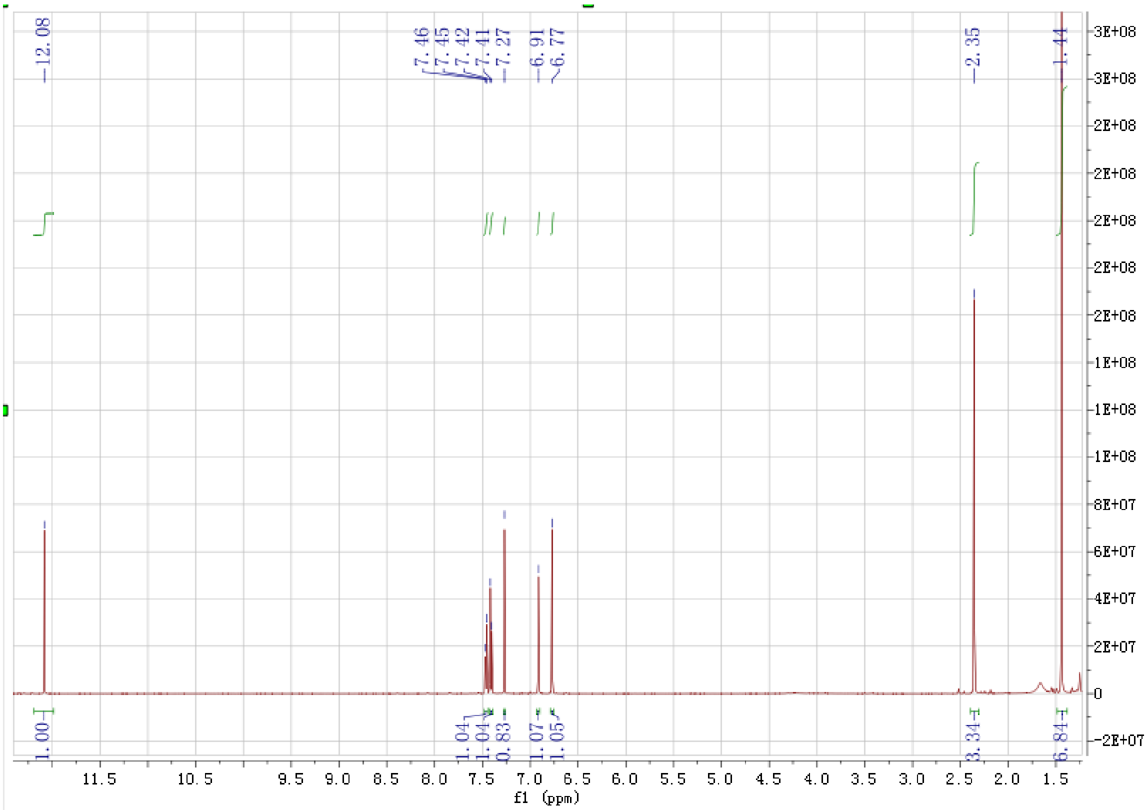

Figure S3. ^{13}C-NMR (125 MHz, $CDCl_3$) spectrum of the new compound acerolanin **A**.

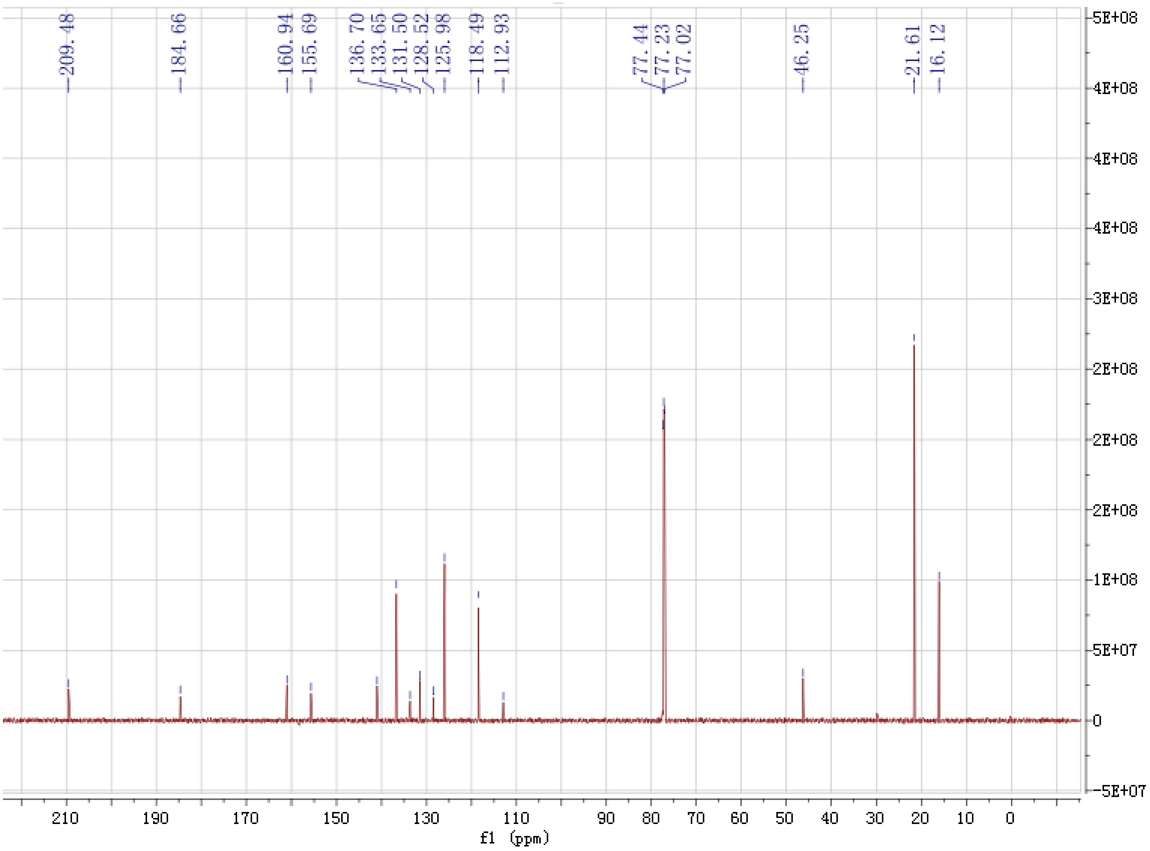

Figure S4. The HMBC spectrum of the new compound acerolanin **A**.

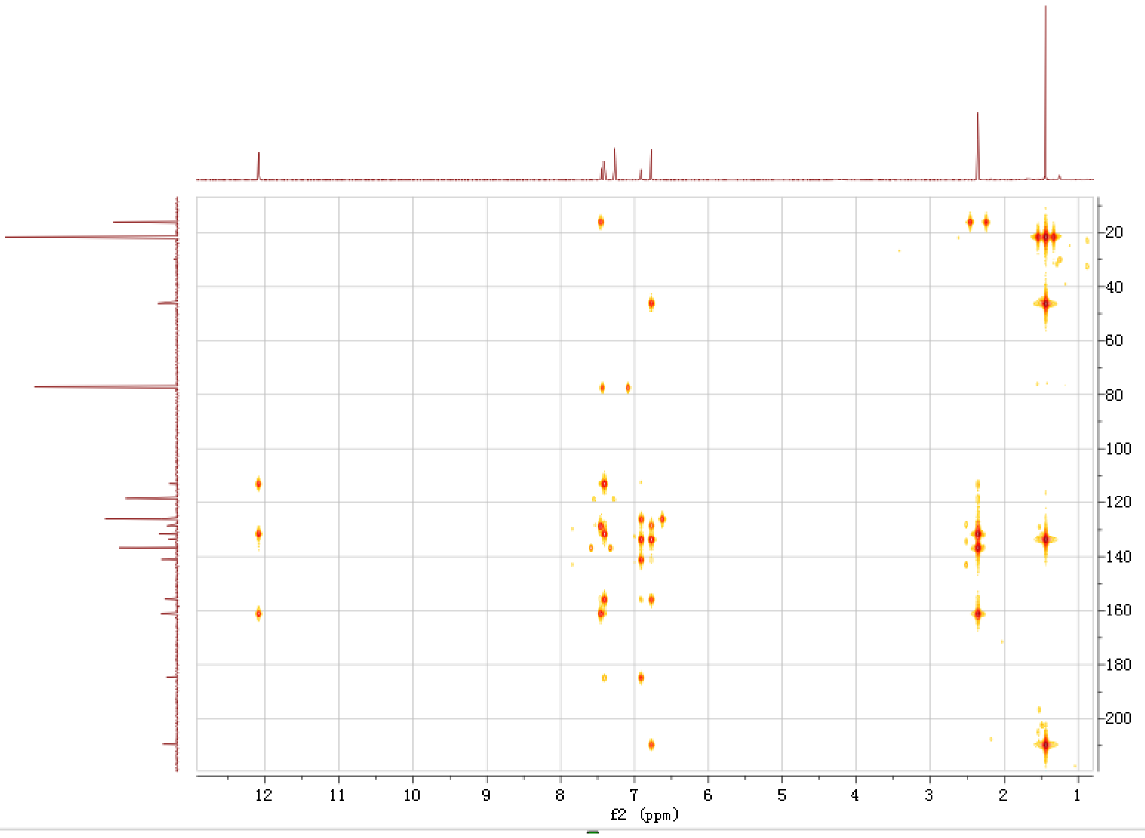

Figure S5. The HSQC spectrum of the new compound acerolanin **A**.

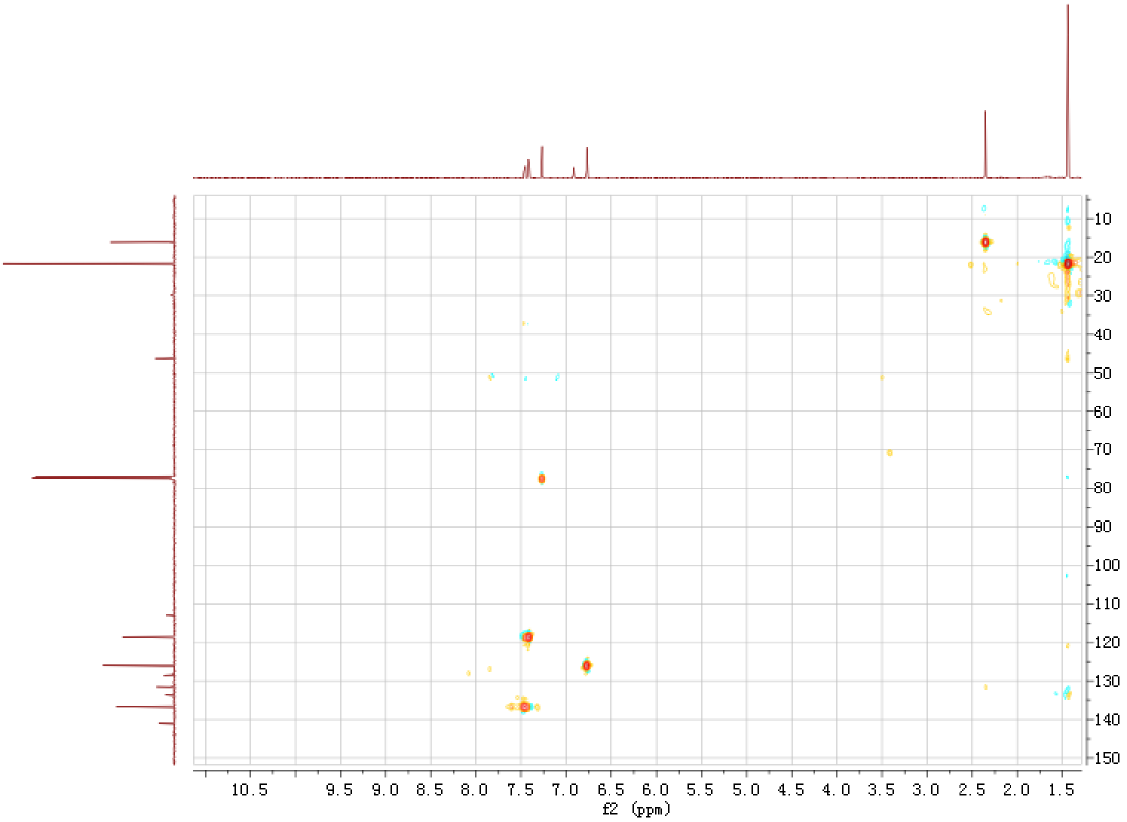

Figure S6. ^{1}H-^{1}H COSY spectrum of the new compound acerolanin **A**.

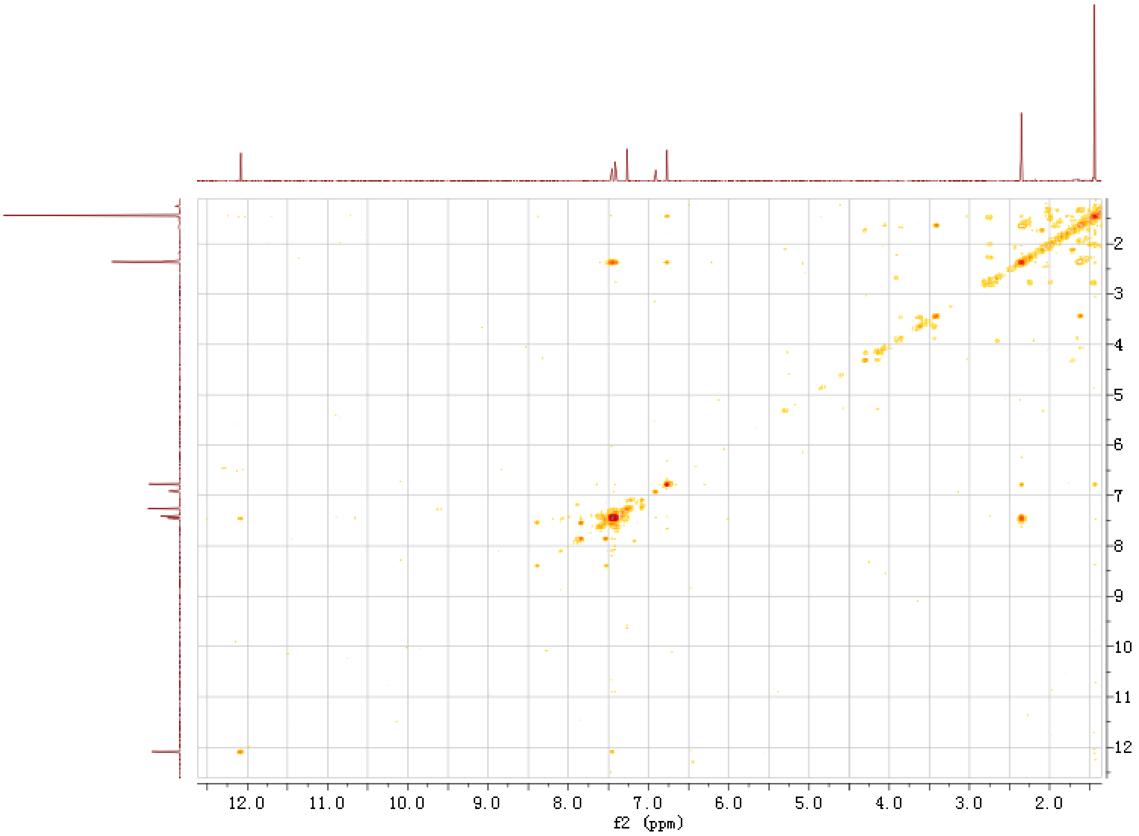

Figure S7. ^{1}H-NMR (600 MHz, $CDCl_3$) spectrum of the new compound acerolanin **B**.

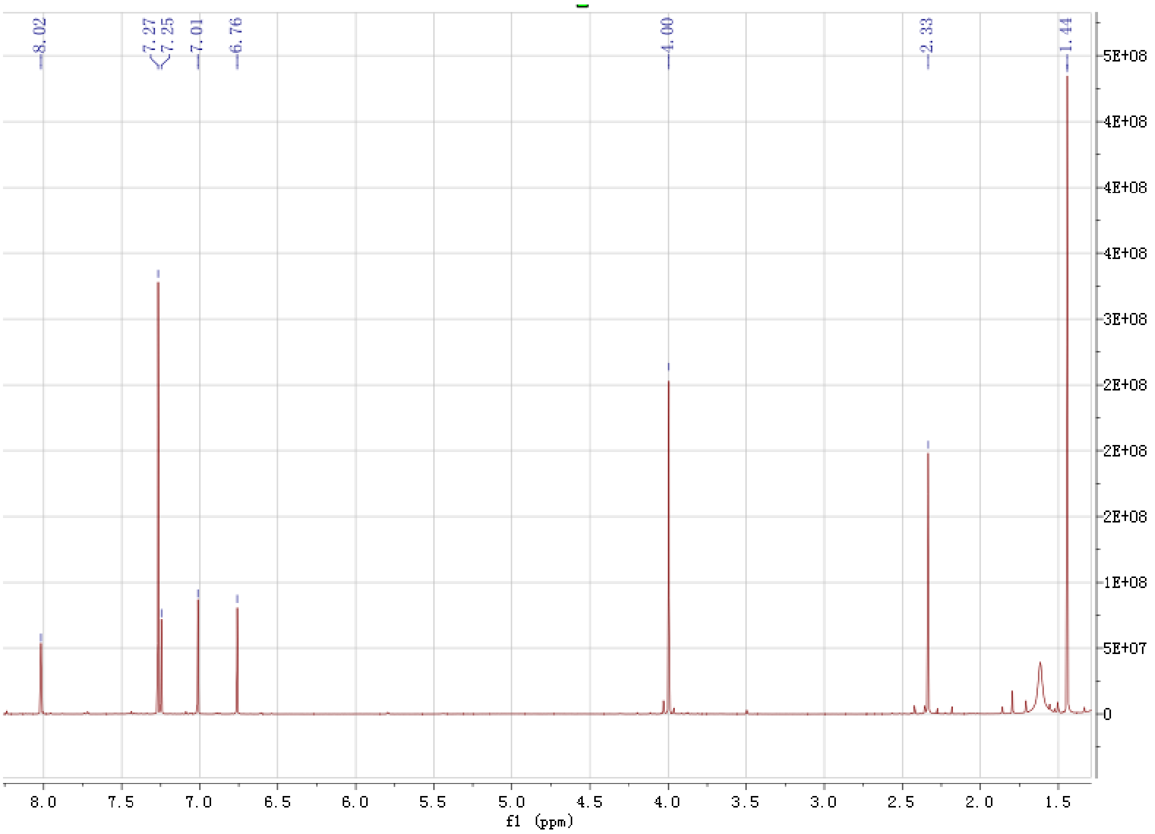

Figure S8. ^{13}C-NMR (125 MHz, $CDCl_3$) spectrum of the new compound acerolanin **B**.

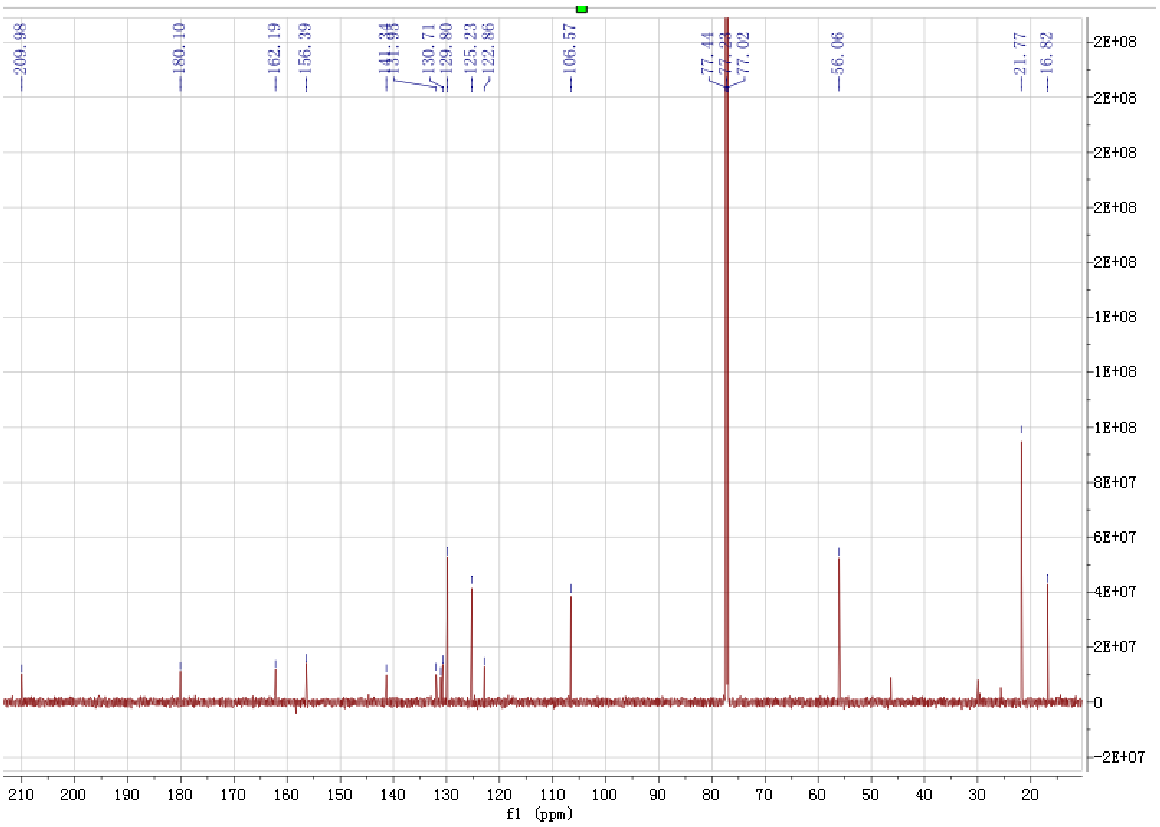

Figure S9. The HMBC spectrum of the new compound acerolanin **B**.

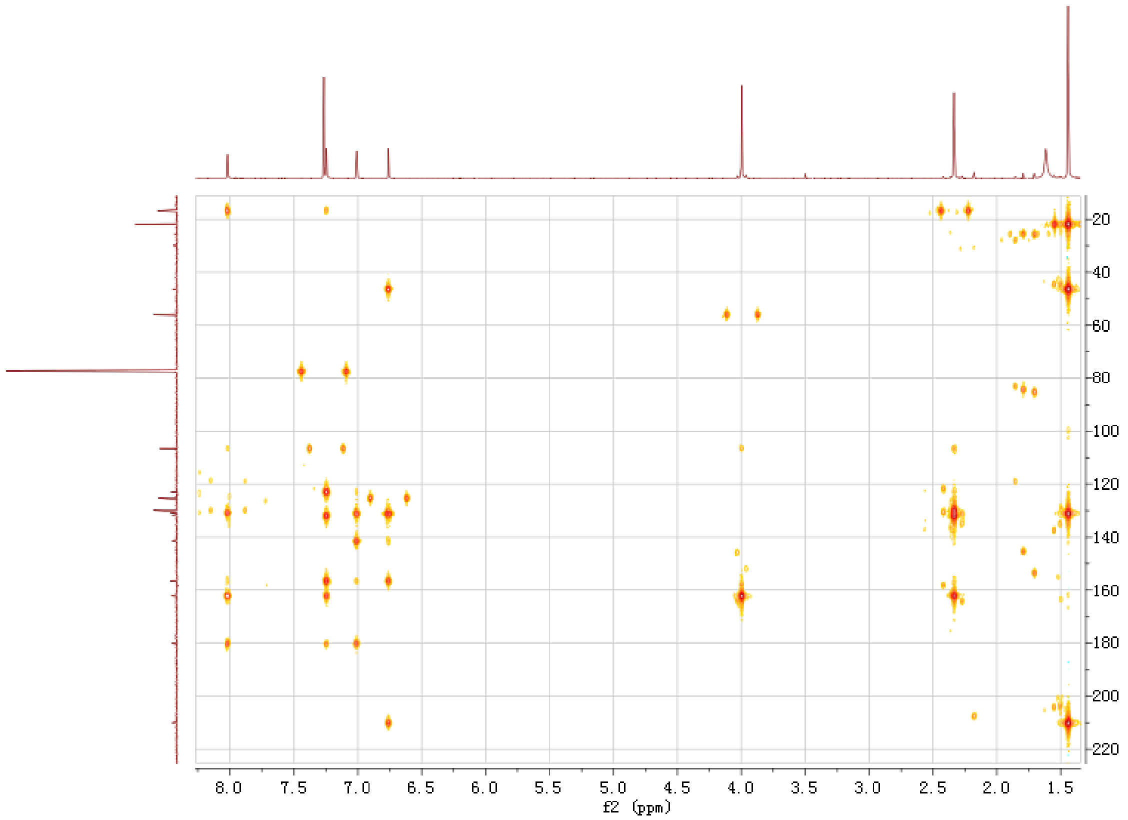

Figure S10. The HSQC spectrum of the new compound acerolanin **B**.

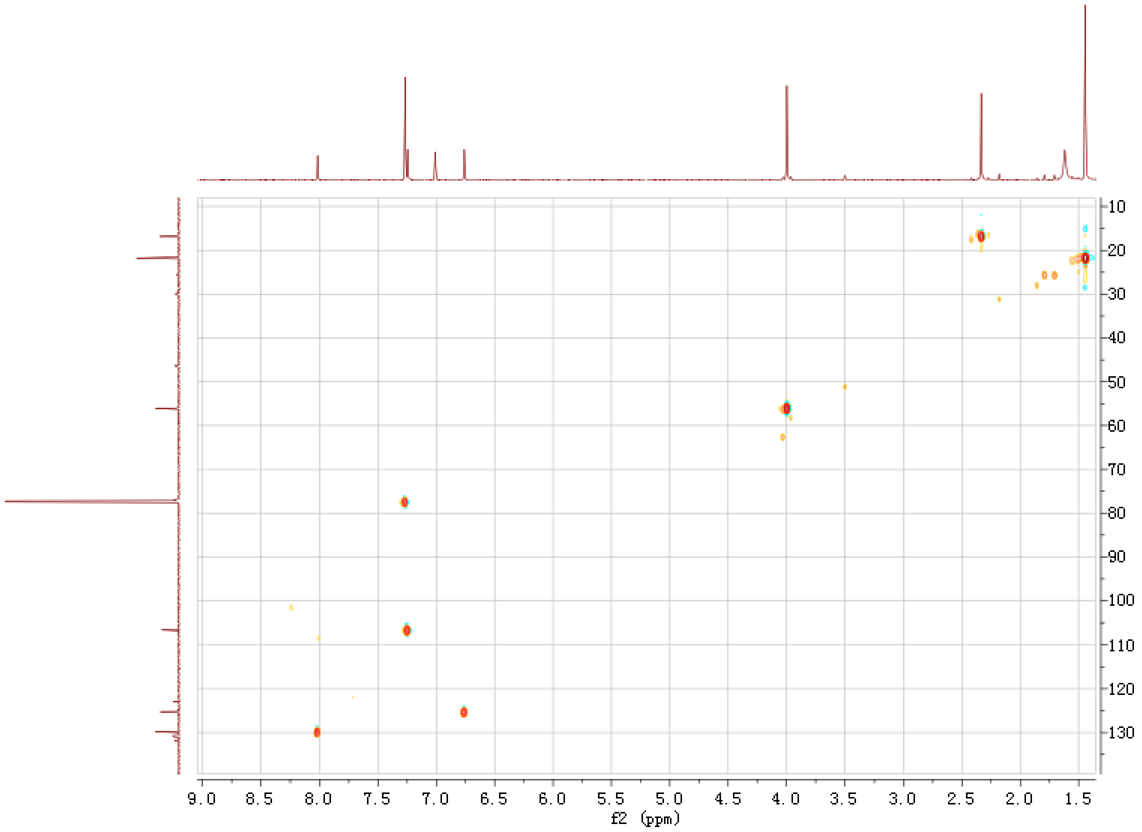

Figure S11. ^{1}H-^{1}H COSY spectrum of the new compound acerolanin **B**.

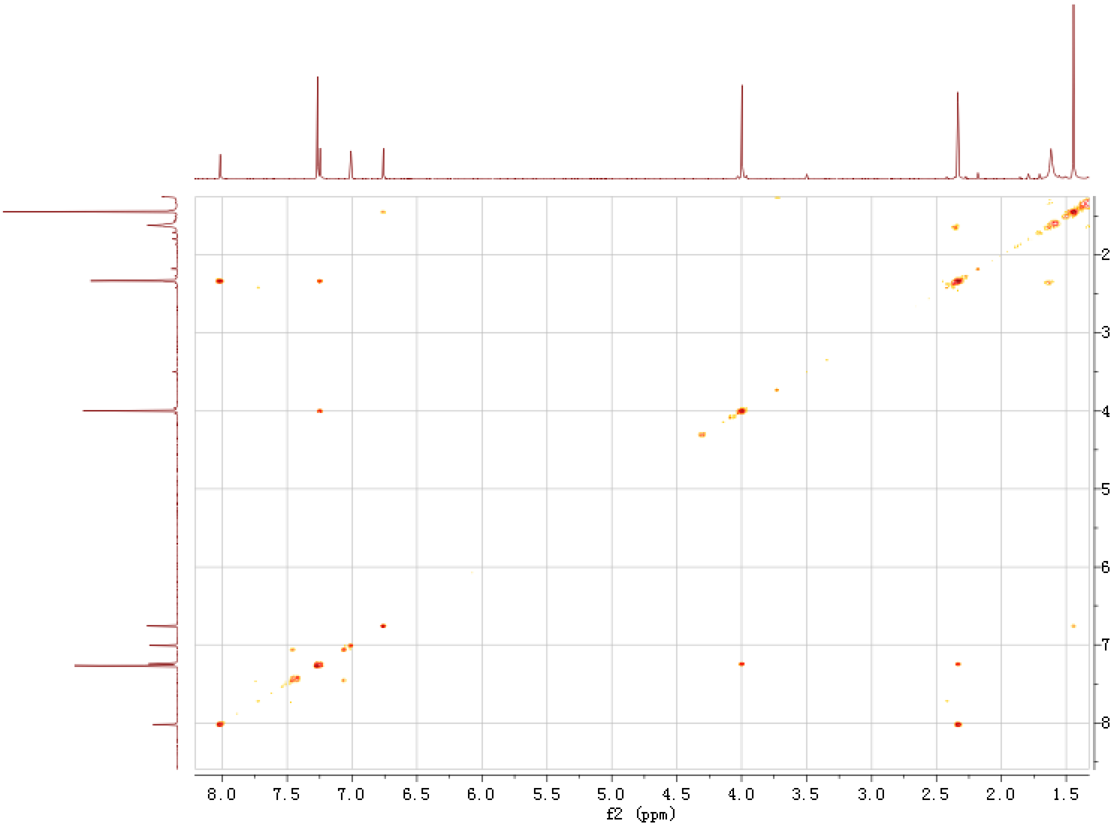

Figure S12. ^{1}H-NMR (600 MHz, $CDCl_3$) spectrum of the new compound acerolanin **C**.

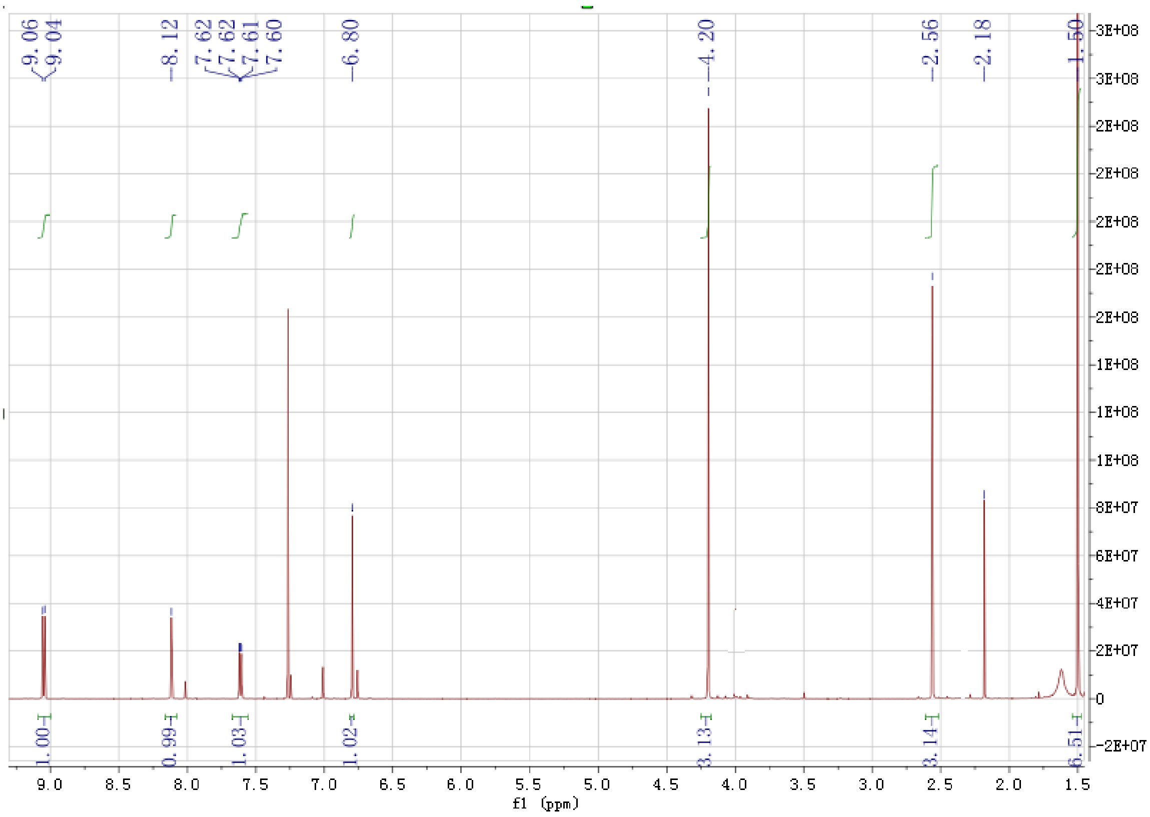

Figure S13. ^{13}C-NMR (125 MHz, $CDCl_3$) spectrum of the new compound acerolanin **C**.

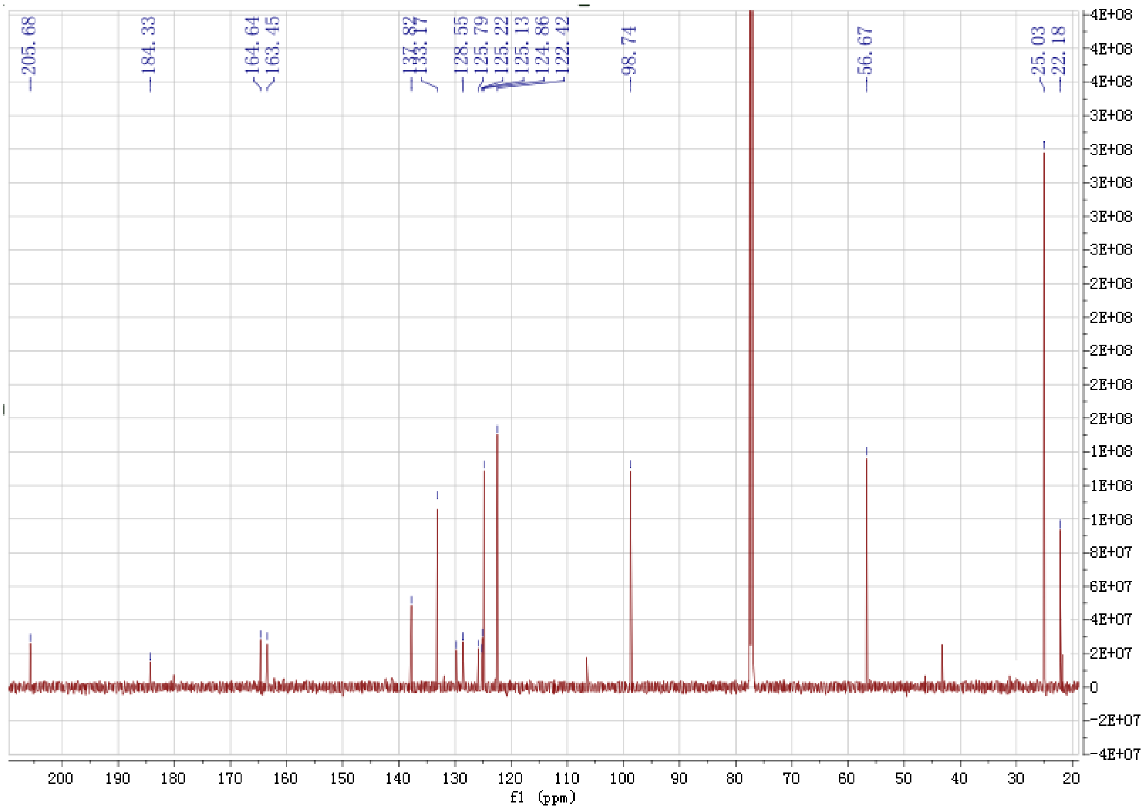

Figure S14. The HMBC spectrum of the new compound acerolanin **C**.

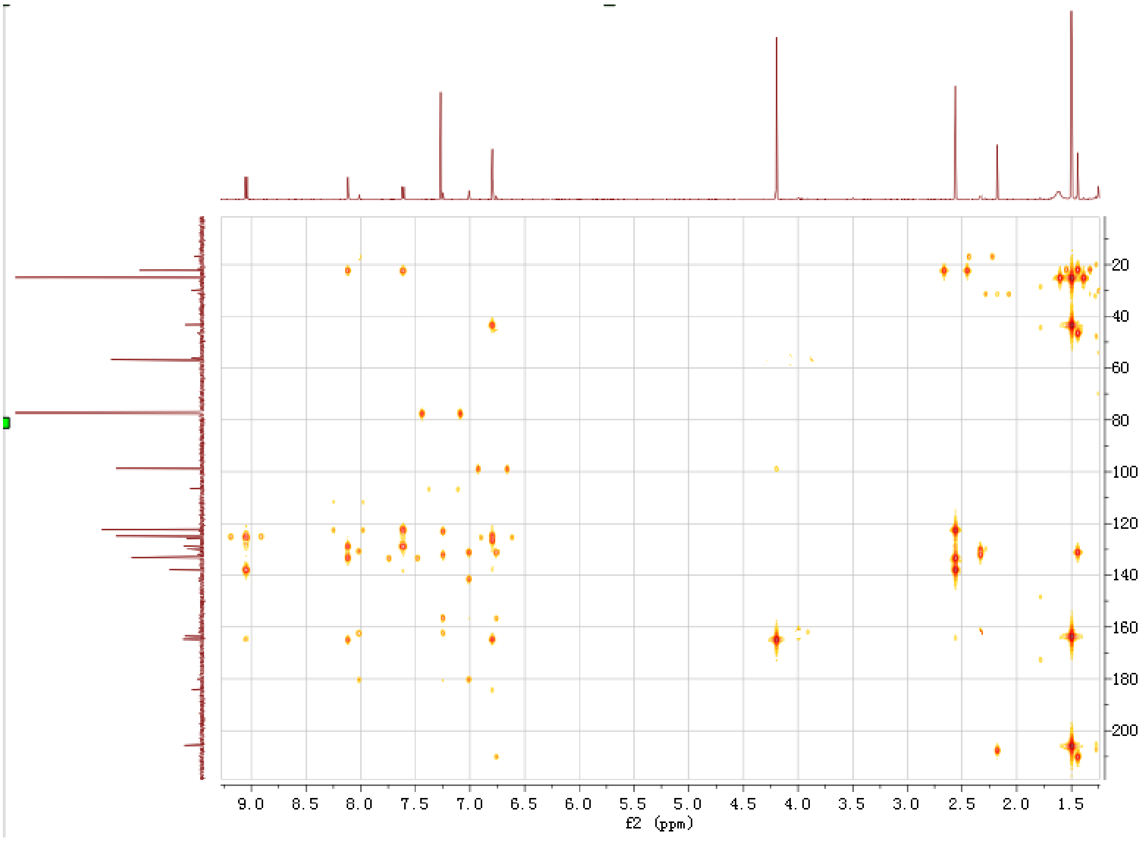

Figure S15. The HSQC spectrum of the new compound acerolanin **C**.

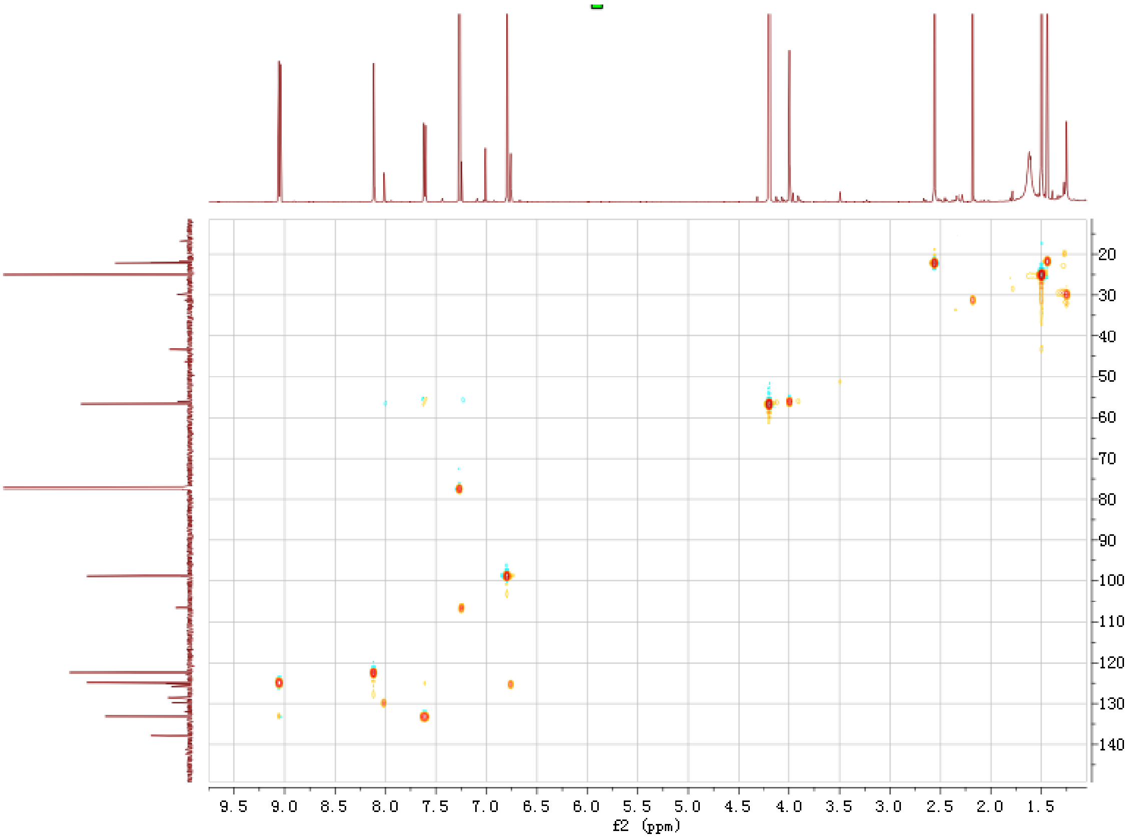

Figure S16. ^{1}H-^{1}H COSY spectrum of the new compound acerolanin **C**.

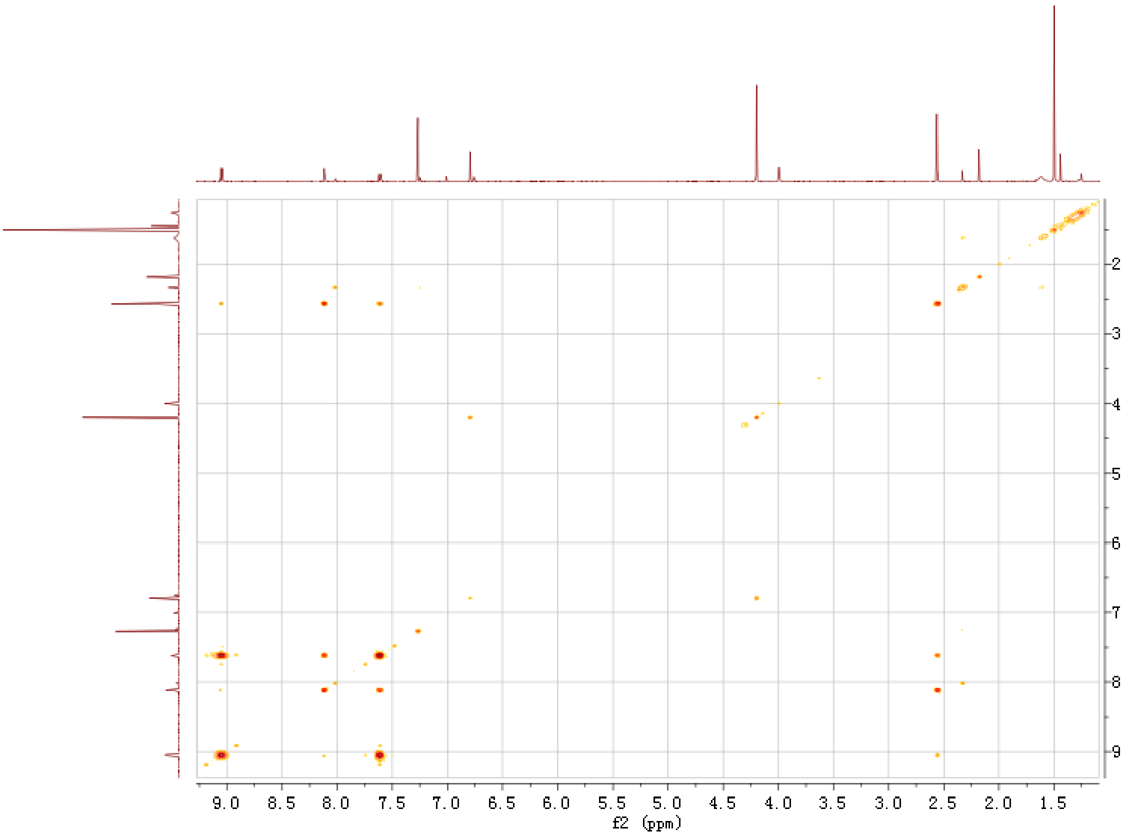

Supplement: Supplementary file 1 [file molecules-19-02629-s001.pdf]
